# Knowledge, Beliefs, and Behaviors Related to Secondhand Smoke and Smoking in the Home: A Qualitative Study With Men in Malaysia

**DOI:** 10.1093/ntr/ntac239

**Published:** 2022-10-14

**Authors:** Raisya Nur Syazmeen Abdul Mutalib, Nurul Latiffah Abd Rani, Aziemah Zulkifli, Norul Hernani Abd Latif, Ruaraidh Dobson, Tengku Azmina Engku Ibrahim, Sean Semple, Emilia Zainal Abidin, Isabelle Uny, Rachel O’Donnell

**Affiliations:** Department of Biomedical Science, Kulliyyah of Allied Health Sciences, International Islamic University Malaysia, Malaysia; Faculty of Ocean Engineering Technology and Informatics, Universiti Malaysia Terengganu, Malaysia; Department of Environmental and Occupational Health, Faculty of Medicine and Health Sciences, Universiti Putra Malaysia, Malaysia; Department of Biomedical Science, Kulliyyah of Allied Health Sciences, International Islamic University Malaysia, Malaysia; Institute for Social Marketing and Health, Faculty of Health Sciences and Sport, University of Stirling, Stirling FK9 4LA, UK; Faculty of Ocean Engineering Technology and Informatics, Universiti Malaysia Terengganu, Malaysia; Institute for Social Marketing and Health, Faculty of Health Sciences and Sport, University of Stirling, Stirling FK9 4LA, UK; Department of Environmental and Occupational Health, Faculty of Medicine and Health Sciences, Universiti Putra Malaysia, Malaysia; Institute for Social Marketing and Health, Faculty of Health Sciences and Sport, University of Stirling, Stirling FK9 4LA, UK; Institute for Social Marketing and Health, Faculty of Health Sciences and Sport, University of Stirling, Stirling FK9 4LA, UK

## Abstract

**Introduction:**

Despite the health risks associated with secondhand smoke (SHS) exposure, smoking in the home is common in Malaysia, and almost exclusively a male behavior.

**Aims and Methods:**

This study explored male smokers’ knowledge, beliefs, and behaviors related to SHS exposure and smoking in the home, to guide future intervention development. Twenty-four men who smoked and lived in Klang Valley, Kuantan, or Kuala Terengganu took part in semi-structured interviews which explored knowledge and beliefs regarding SHS in the home, and associated home-smoking behaviors. Data were managed and analyzed using the framework approach.

**Results:**

There was limited knowledge regarding the health risks associated with SHS: the smell of SHS in the home was a more prominent concern in most cases. Many had no rules in place restricting home smoking, and some suggested that smoking in specific rooms and/or near windows meant SHS was not “shared” with other household members. A few fathers had created but not maintained a smoke-free home prior to and/or after their children were born. Desire to smoke in the home conflicted with men’s sense of responsibility as the head of the household to protect others and set a good example for their children.

**Conclusions:**

Men’s home-smoking behaviors are shaped by a lack of understanding of the health risks associated with SHS exposure. Gaining a broader understanding of the factors that shape men’s decisions to create a smoke-free home is important to facilitate the development of culturally appropriate interventions that address their responsibility to protect other household members from SHS exposure.

**Implications:**

Our findings highlight the need for public information campaigns in Malaysia to educate men who smoke regarding the health harms associated with SHS in the home and the ways in which SHS travels and lingers in household air. This is important given men’s concerns about SHS often focus on the smell of cigarette smoke in the home. Our findings suggest a number of potential avenues for future intervention development, including household and community-level initiatives that could build on men’s sense of responsibility as the head of the household and/or their general desire to protect their families.

## Introduction

Malaysia has a relatively high adult smoking prevalence compared with other South Asian countries, almost one-quarter (21%) of Malaysian adults are smokers.^1^ Smoking is almost exclusively a male behavior, with 43% of men aged 15 years and over reported to smoke compared with 1.4% of women.^2^ The Malaysian Government has adopted a number of measures to reduce the risks of secondhand smoke (SHS) exposure, prohibiting smoking in several types of public places and workplaces,(for example ^3,4^) in line with the Framework Convention on Tobacco Control (FCTC), ratified by the Malaysian government in December 2005. The FCTC encourages signatory countries to implement universal measures to protect nonsmokers from SHS exposure, and to ensure at least 90% of their population are protected from SHS exposure through smoke-free policies or laws.^5^ However, in Malaysia compliance with smoke-free legislation, is reported to be relatively low.^6^

There is no safe level of exposure to SHS, which has been shown to have wide-ranging adverse health effects on adults and children, including lower respiratory infections and asthma, ischemic heart disease, and lung cancer.^7,8^ In Malaysia, approximately 1 in 4 (25.9%) nonsmoking adults report being exposed to SHS in the home, with exposure rates higher among females (31.3%).^9^ In a recent study of 420 pregnant Malay women 95% of the 209 who reported being exposed to SHS were exposed at home, as a result of their husband smoking.^10^

Few studies have reported on the proportion of children reporting SHS exposure in the home in Malaysia. A cross-sectional study conducted in 2011 measured salivary cotinine concentrations among 1064 school children (aged 10–11 years). This study found that 52.9% were exposed to SHS at home,^11^ a much higher figure compared to the global estimate of 40% of children exposed to SHS.^12^ A more recent study used self-report questionnaires with children aged 10–11 years (*n* = 312) living in the rural area of Kelantan. The prevalence of children’s SHS exposure at home was 55.8%, with nearly half of children (44%) living in a home with two or more adults who smoked.^1^ Only 22% of Malaysian adult smokers and 47% of Malaysian adult nonsmokers report having a completely smoke-free home, according to 2015 Global Adult Tobacco Survey (GATS) data.^13^

There is little published research examining SHS awareness and understanding in Malaysian adults. A recent secondary analysis of GATS data suggested that age, education, and ethnicity are influencing factors. Older individuals (≥65 years) were found to be less aware of the effects of SHS than younger individuals (15–24 years). Less well-educated individuals were less aware of the effects of SHS than those with higher education levels, and Malay individuals had higher SHS awareness levels compared with individuals of Chinese, Indian, or “other” ethnicity. The sample comprised predominantly nonsmokers (82%), though the proportion of men (49%) and women (51%) surveyed was similar.^14^ Parental health perceptions and preventive measures related to children’s SHS exposure were examined in a recent cross-sectional nationwide study.^15^ Most respondents were mothers (76%) and nonsmokers (87%). Although most were aware of the health risks associated with children’s SHS exposure, almost one-third (35%) had no smoke-free home (35%) or car (32%) rules in place. Most (85%) had instead taught their children to stay away from smokers. Parents (fathers) who smoked often allowed smoking in the home with an open window, and they viewed children’s exposure to SHS as less risky compared to nonsmoking parents.^15^

Several published papers have included calls for the development of policies, campaigns, and interventions to reduce nonsmokers' and children’s exposure to SHS in homes in Malaysia.^1,9,11,14–16^ It has been suggested that in some Asian cultures traditional gender-based norms may cause some women to refrain from challenging men in their households when they are smoking in the home, even when women perceive the risks of SHS to be high for their children.^17,18^ An enhanced understanding of adult male smokers’ knowledge, beliefs, and associated home-smoking behaviors could inform the development of behavioral change and educational interventions targeted at men. This is important given the international smoke-free homes literature is dominated by interventions focusing on the role of women and mothers,^18,19^ and there is no consensus on the features that define “effective” smoke-free home interventions.^20^ On this basis, the aim of this qualitative study was to explore adult male smokers’ knowledge, beliefs, and behaviors related to SHS exposure and smoking in the home, to inform future intervention development.

## Methods

In line with recent calls for capacity development in global health studies,^21,22^ we embedded qualitative research capacity building within this study, to equip the Malaysia study team with the skills and confidence to independently conduct qualitative studies in the future. RNSAM, AZ, and NLAR had no prior experience in conducting qualitative research. Along with their supervisors (NHAL, TAEI, and EZA), they engaged in 13 capacity-building sessions across the 18-month study, facilitated by RO and IU, two members of the UK study team who are experienced, qualitative researchers. These sessions included bespoke training on reflexivity, the development of topic guides, interviewing skills and techniques, online, face-to-face, and telephone interviewing and the use of NVivo 12 to support coding and framework analysis. In addition, regular qualitative team meetings facilitated reflection, discussion, and problem-solving during fieldwork and analysis. Our reflections on the practicalities, challenges, and advantages of conducting qualitative research capacity building online during the coronavirus disease 2019 (COVID-19) pandemic will be published separately.

### Recruitment

The inclusion criteria were: (1) an adult (>18 years old), male, and current smoker (combustible cigarettes) who smoked indoors at home, and (2) living in a home with at least one child under the age of 16 years. Individuals were recruited to the study through visits to local public places including restaurants, shopping malls, and kindergartens (*n* = 4), where the study was discussed and participant information sheets were distributed. Individuals were also recruited using Facebook and Instagram advertisements and WhatsApp messages (*n* = 20). All recruitment targeted the three geographical areas of Peninsular Malaysia; Klang Valley (Selangor), Kuantan (Pahang), and Kuala Terengganu (Terengganu). Individuals interested in participating were invited to submit their contact details securely online and confirm eligibility to participate. Those recruited via social media were then sent the participant information sheet and consent form to complete via WhatsApp in advance of interviews being arranged. Where face-to-face interviews were conducted, consent forms were completed at the start of each arranged interview.

### Qualitative Interviews

Using a convenience sampling strategy, 24 men consented to participate and were interviewed between January and August 2021. Interviews were conducted by telephone (*n* = 19), online video call (*n* = 1), or face-to-face (either at the University or in the participant’s home [*n* = 4]) depending on participant preference and in line with COVID-19 guidelines in place at the time. The semi-structured interviews were conducted by RNSAM, AZ, and NLAR, female members of the research team. Interviews lasted approximately 30 minutes and were audio-recorded with participant permission. The interview topic guide included an exploration of smoking history, smoking rules in the home, beliefs about SHS, barriers, and facilitators to creating a smoke-free home and perceptions of other family members’ views on smoking in the home. Each interviewee was gifted a voucher worth RM50 (£9) for their time, travel, and participation, in line with current practice in the three Malaysian Universities involved. Ethical approval was granted by the University of Stirling General University Ethics Panel [GUEP (1920) 940] and the Ethics Committee for Research Involving Humans Subjects of Universiti Putra Malaysia (JKEUPM) [JKEUPM-2020-393].

### Qualitative Analysis

Interviews were transcribed and translated into English from Malay by RNSAM, NLAR, and AZ, using professional transcription companies in each local area. Transcripts were not provided to participants for approval prior to translation. Anonymized transcriptions were uploaded into NVivo 12 for coding. Two transcripts were initially coded by RNSAM, AZ, and NLAR during two online training sessions facilitated by RO and IU to support initial skills development. RNSAM, AZ, and NLAR then independently coded one transcript each, and during a third online session, we discussed and resolved any areas of discrepancy. RNSAM, AZ, and NLAR conducted further coding independently, with the regular support, discussion, and input. Transcripts were then analyzed (by RNSAM, NLAR, and AZ) using the framework approach^23^ alongside the use of memos to support reflexivity.^24^ A thematic framework was developed (by RO, IU, RNSAM, AZ, and NLAR) to guide data analysis, using deductive (considering the topic guide) and inductive (reading transcripts and coding) techniques. To prepare for detailed analysis, data summaries were written in relevant cells of the framework grid (RNSAM, AZ, and NLAR), including hyperlinks to transcripts to facilitate data retrieval. RO and IU reviewed all summaries to check the consistency of approach and interpretation of the data. Data summaries were then used to identify high-level themes (RNSAM, AZ, NLAR, RO, and IU) before further in-depth analysis was conducted. Themes were finalized based on reexamining data and reflexive team discussions (RNSAM, AZ, NLAR, NHAL, RO, and IU).

## Results

Participant characteristics are presented in Supplementary File 1. They were 26–69 years old (mean age 36 years). Twenty-one were fathers, one was an expectant father living with young nephews and two were adult-aged sons living with parents and siblings. Seventeen lived in households from the lowest 40% (B40) of the Malaysian national income category (mainly those recruited in Terengganu and Pahang), and seven lived in households representing the middle 40% income bracket (M40), based on the Department of Statistics Malaysia (DOSM) income classification system. All were of Malay ethnic origin. Themes are presented alongside illustrative quotes below, alongside each participant's ID number and their home-smoking arrangements. Men’s accounts were compared and contrasted and nuanced differences observed in relation to income level and men's knowledge and beliefs about SHS are also presented below.

### Men’s Knowledge and Beliefs About SHS

#### Perceptions of Risk Associated With SHS Exposure in the Home

Interviewees held a range of views on the extent to which SHS exposure in the home constitutes a health risk. Some men, and in particular those in the middle household income group, were aware of health risks including lung cancer, heart disease, and other respiratory impacts. A few also noted that babies and children are particularly at risk from SHS exposure, with one father saying:

*“Of course the breathing…I’ve read that…because children, especially children who are under teen-age or something like that, their lungs are still small.”* (Participant 20, smokes in the bathroom but considers they have a smoke-free home).

In contrast, several men from lower-income households seemed unclear about specific health risks associated with SHS exposure, sharing views that focused on relative risks. Some suggested that SHS exposure is more harmful than active smoking, because active smokers inhale smoke through a cigarette filter which reduces harm:

“*They inhale our cigarette smoke without a filter. It is more dangerous. [That’s what] Others tell me.”* (Participant 10, No rules in place restricting smoking in the home).

A few perceived SHS exposure to be far less of a risk to health than outdoor air pollution:

*“For me, cigarette smoke and the factories that... release smoke...the factories are worse [than SHS exposure]. If I wanted to calculate [the risks]…Millions times more.”* (Participant 2, no rules in place restricting smoking in the home).

One participant suggested that smoking in the home has no impact on other household members, saying: *“it doesn’t directly affect others.”* (Participant 4, no rules in place restricting smoking in the home).

#### Concerns Regarding the Smell of SHS in the Home

When asked about their views on SHS, most men initially voiced concerns related to the smell of cigarette smoke in their homes, and this seemed to be a more salient concern for several interviewees than the health effects of SHS exposure. Several noted that other household members disliked the smell of SHS in their homes, and in some (but not all) cases men were persuaded to smoke elsewhere as a result. One father explained he was often *“told to smoke in other areas* (of the home) *because of the smell”* (Participant 15, no rules in place restricting smoking in the home). Another suggested:

[it is] *“better [to] smoke outside of the house so that everyone in the house will not be talking about the smell of the smoke”* (Participant 21, no rules in place restricting smoking in the home).

Another father stated that although his child often complained about the smell of smoke in the home, he continued to smoke in the living room. When asked why he was concerned about SHS, he replied: “*Because inside the house [after smoking], then the smell is bad.”* (Participant 13, smoking is allowed in the living room).

In other cases, men made efforts to mask the smell of smoke in the home, because they did not want to create a bad impression when visitors entered their homes. One father described failed attempts to try to mask the smell of cigarette smoke in his house in this way:

*“If guests come over, ya the first thing they will detect is the cigarette smell. Even if I buy [air freshener brand name]...but I still keep on smoking, the fragrance will not make any difference.”* (Participant 7, Smoking is allowed in the home, but not in front of others).

### Men’s Indoor Smoking Behaviours

#### Smoking Locations

Half (*n* = 12) of interviewees reported no rules in place restricting smoking in the home (see Supplementary Table 1), and several had never attempted to introduce these rules. Seven had partial smoke-free home rules—smoking only in specific rooms (the bathroom, living room, and/or kitchen), or smoking in the home only when other household members were not present. Men often chose to smoke near open windows in an attempt to keep SHS out of the house (“The room has windows, and when I smoke I exhale the cigarette smoke outside.” [Participant 1, smoking is allowed in the bedroom and the loft]). Five men reported that smoke-free home rules were in place, however in each case during the course of the interview, this was contradicted by references made to smoking indoors. One father justified this by saying that although he smoked in the bathroom, “that means I don’t smoke openly in the house” (Participant 5, smokes in the bathroom but considers they have a smoke-free home), suggesting a limited understanding regarding the ways in which SHS travels and remains suspended in the air.

#### Efforts to Contain or Conceal Smoking in the Home

A few men spoke of choosing to smoke in the home when no other household members were present, and/or trying to minimize or conceal smoking from other family members and visitors. In some cases, this provided a rationale for smoking in the bathroom “Oh, that’s my favorite spot” (Participant 7, Smoking is allowed in the home, but not in front of others), which was considered a private space by many men. A strong desire to keep smoking away from children was evident in a few fathers’ accounts, generally to “*set a good example*” (Participant 15, No rules in place restricting smoking in the home). As one father noted:

“*Every time I smoke outside…ya they [children] might see [me], snooping through the windows to see [me]. But I don’t show taking out a cigarette in front of them, lighting it. That’s how it is. As much as I can, I avoid it* [smoking] *in front of the children.”* (Participant 4, no rules in place restricting smoking in the home).

However, in some other cases, interviewees acknowledged smoking in front of other household members, for example in the living room whilst watching television with their wife and/or children and in these instances, SHS exposure did not appear to be a concern:

*“Yes, depending on the situation. Sometimes I still smoke even when the kids are in the house [laughs]”* (Participant 8, Smoking is allowed in the living room).

### Factors That Influence Home-Smoking Behaviors

#### Protecting Young Families

Several interviewees had never attempted to create a smoke-free home before, and some suggested this could only be achieved by quitting smoking completely “if the smoker wants to make his home smoke-free, he needs to stop smoking first” (Participant 1, smoking is allowed in his bedroom and the loft). However, a few fathers gave retrospective accounts of creating but not maintaining a smoke-free home, at times when other household members were considered particularly vulnerable to the health effects of SHS exposure. One father reported creating a smoke-free home during his wife’s pregnancy, explaining that “I respect my wife, in that situation, you know…I know (SHS exposure) is not good for humans.” His decision to resume smoking in the home again was guided by his perception that as his babies grew into young children, they were less susceptible to SHS harms:

*“My children were already running about...have grown up... I assumed it* [smoking in the home] *will be alright.* (Participant 7, Smoking is allowed in the home, but not in front of others).

Another father who had created a smoke-free home once his child was born described his inability to maintain this change in the months that followed, which he attributed to his own failings:

“*When my child was three to four months old, I smoked cigarettes in the kitchen…I can’t change my smoking habits because I’m lazy to leave the house. Smoking is very dangerous...to the children and others.*” (Participant 10, No rules in place restricting smoking in the home).

In contrast to these retrospective accounts, we did not observe any patterning of reported smoke-free home rules by the number/age of children in the household (see Supplementary File 1).

#### Habitual Behaviors Versus Responsibilities as Head of the Household

“Laziness,” “habit,” and “stubbornness” were mentioned by a few other interviewees as a barrier to creating a smoke-free home (Participant 11, no rules in place restricting smoking in the home; Participant 23, Smoking is allowed in some rooms). Their desires and impulses to smoke in the home often conflicted with the sense of responsibility they acknowledged as the head of the household to set a good example to family members. Under Islamic law in Malaysia, smoking is classified as an activity that is strictly forbidden, and in a few instances, fathers recalled children discouraging them from smoking on this basis:

*“’Smoking is bad,’ ‘Cigarette is sinful’* [laughs]*, ‘God said you cannot smoke’ – those are some of what [my] children would say”* (Participant 20, smokes in the bathroom but considers they have a smoke-free home).

Keeping smoking out of sight by smoking outdoors was also acknowledged by a few fathers as a possible means of reducing the likelihood that other family members would take up smoking in the future:

*“If the head of the family sets a bad example to the family members, then they will also follow this bad thing [smoking]”* (Participant 14, No rules in place restricting smoking in the home).

#### Respect for/Support From Family Members

Several fathers also spoke of the importance of respecting other household members and taking steps towards creating a smoke-free home was often discussed in this context. Some interviewees suggested that their wives played an important role in reminding and/or supporting them to smoke in other areas of the home, or outside. One father noted that his wife had assisted him to create an outdoor smoking area, which he now used some of the time:

*“My wife plays an important role to make the home smoke-free. For example, my wife bought a hammock…I built a gazebo as a station [pause] for smoking area. So, I go to smoke there”* (Participant 9, smokes in the kitchen but considers they have a smoke-free home).

In one case the pursuit of cleanliness and freshness in the home was viewed as integral to protecting the health of family members from SHS in the home:

*“The solution is that we must think that this is our house. It should be a clean space with a nice smell, a calm area…so, it won’t affect the health of child and wife. So, we should not take it easy on this matter [of smoking in the home].”* (Participant 17, smoking is allowed in the kitchen and bathroom).

## Discussion

The current study explored adult male smokers’ knowledge, beliefs, and behaviors related to SHS exposure in the home in Malaysia. Men often had limited knowledge regarding the health risks associated with SHS, and the smell of SHS in the home was a more prominent concern in most cases. Half of the men interviewed reported no rules in place restricting home smoking, and some suggested that smoking in specific rooms (in particular in the bathroom) and/or near open windows meant that SHS was not “shared” with others in the household, a finding also reported in qualitative research conducted with fathers/grandfathers who smoke in China.^25^ This suggests that inaccurate beliefs and/or incomplete knowledge about SHS can contribute to exposure among other household members. Public health information campaigns are required to educate men who smoke about the health harms associated with SHS exposure in the home, and the way in which SHS travels and lingers in the household air for a considerable period after smoking a cigarette.^26^ This information could also be used in interventions to encourage men to make their home smoke-free, alongside personalized measurement of home air quality, which provides an accurate and noninvasive proxy measure of SHS exposure and has been shown to be effective in reducing SHS levels in the home in Global North settings.^27^ Additional work conducted as part of this study suggests this approach also shows promise in a Malaysian context.^28^

Our finding that the smell of SHS in the home was a prominent concern for most men may in part reflect the emphasis that the Islamic faith places on physical and spiritual cleanliness. These findings are also important given recent suggestions that some of the world’s largest tobacco companies are now marketing “less smoke smell” (LSS) cigarettes in low and middle-income countries.^29^ Terms used to communicate LSS including “reduced room smell of tobacco smoke” and “less smell on and around you” may increase social acceptability of smoking.^29^ They may also appeal to smokers who are concerned about the smell of cigarettes in their home, especially in households where the health risks associated with SHS exposure are poorly understood. This also highlights the importance of developing information campaigns to educate men that the health harms associated with SHS exposure are not lessened by reduced-odor cigarettes. Such information would better equip smokers to change home-smoking behaviors, alongside recent calls for Governments to introduce policies to restrict LSS labeling claims.^29^

Several men expressed a general desire to protect others from SHS exposure in the home, noting their sense of responsibility as the head of the household to set a good example to their children and other household members. Whilst these values were not often reflected in their home-smoking rules, this “father-protector” role, often interlinked with the notion of being a “good” father, has been found to be a key motivator for reducing children’s home SHS exposure in recent research conducted in Canada and Scotland.^30–33^ Community-based smoke-free home initiatives may be effective in facilitating such shifts. One recent pilot initiative in Kerala, India used a combination of healthcare worker household visits, and an educational video with positive messages to support fathers’ abstinence from smoking in the home as a sign of caring for women and children, and as a social value linked to the cultural value of male responsibility.^34^ This approach could be usefully explored in a Malaysian context. Whilst previous research suggests that in some Asian cultures women may refrain from challenging men in their households when they smoke in the home, ^17,18^ this did not appear to be the case in our study. Some men reported their wives’ had an active role in persuading them to smoke elsewhere in the home and in one case supporting them to create an outdoor smoking environment. Further research could explore men's and women’s roles in more detail with a view to co-creating a family-based smoke-free home intervention. Household-level interventions would harness existing partner support for changing home-smoking behaviors and the involvement of wives could be especially important given nonsmoking parents in Malaysia have been found to view SHS exposure to children as riskier than smoking parents.^15^

We are not aware of other Malaysian studies that have utilized a qualitative approach to explore the issue of home smoking and the effect of SHS. Our findings present new insights that can form the basis for developing interventions to actively involve and appeal to fathers and other male household members, and health promotion messages on home smoking. Most interviews were conducted online or by telephone during the Movement Control Order (MCO) that was implemented in the early phase of the COVID-19 pandemic. In a few cases, children were present at some stage during the interview, which may have impacted on the data collected in a few instances where fathers felt less comfortable discussing their smoking behaviors in front of their children. As a team, we discussed the possible dynamics of female researchers conducting interviews with men (see, for example^35^), and the possible complexities of female researchers interviewing men about their smoking behaviors, given smoking is almost exclusively a male behavior in Malaysia. RNSAM, AZ, and NLAR kept reflexive logs during fieldwork and their collective experience suggests that men were generally comfortable speaking openly with them about their smoking behaviors, taking time to respond to questions and engage in emotional reflection.

We endeavored to recruit a range of ethnic groups for this study, including Chinese and Indian, however, the men who took part were all Malay. Our findings do not represent the views of male smokers in other ethnic groups. Future qualitative research in this area would benefit from wider reach, especially as quantitative data has suggested that Chinese individuals are less likely to be aware of the effects of SHS compared with Malay and Indian individuals.^14^ In addition, whilst a few fathers proactively spoke about smoking in relation to their Islamic faith, we did not proactively explore men’s feelings about smoking in this context. Work in other countries, including in Bangladeshi- and Pakistani-origin Muslim communities has suggested that religion is an important determinant of beliefs and attitudes towards smoking^36–38^ This would be an interesting area to explore using qualitative research in Malay communities in the future.

## Conclusion

Men’s current home-smoking behaviors are shaped by a lack of knowledge and understanding of the health risks associated with SHS exposure. Gaining a broader understanding of the factors that shape men’s decisions to create a smoke-free home is important to facilitate the development of culturally appropriate interventions that address their responsibility to protect nonsmoking household members and children from SHS exposure.

## Supplementary Material

A Contributorship Form detailing each author’s specific involvement with this content, as well as any supplementary data, are available online at https://academic.oup.com/ntr.

ntac239_suppl_Supplementary_File

ntac239_suppl_Supplementary_Taxonomy-form

## Data Availability

The anonymized data underlying this article may be shared on reasonable request to the corresponding author following review.
