# Supplementary material for: Knowledge, Beliefs, and Behaviors Related to Secondhand Smoke and Smoking in the Home: A Qualitative Study With Men in Malaysia
Source: Nicotine Tob Res. 2022 Oct 14;25(4):821–7. doi: 10.1093/ntr/ntac239 (PMC10032199; doi:10.1093/ntr/ntac239)
Supplement: ntac239_suppl_Supplementary_File [file ntac239_suppl_supplementary_file.docx]

Supplementary file 1: Characteristics of study participants

| Participant | Role in household | Children in the household | Education Level* | Household status** | Age | House Type | Home smoking rules | Other Home Smokers |
| --- | --- | --- | --- | --- | --- | --- | --- | --- |
| 01 | Adult son | Lives in a household which includes a 14 year child | Middle | B40 | 25-34 | Bungalow | Smoking is allowed in his bedroom & the loft | 1 (father) |
| 02 | Father | 6 (age 4 to 24) | Low | B40 | 35-44 | Terrace | No rules in place restricting smoking in the home | 1 (son) |
| 03 | Adult son | Lives in a household which includes a 1 year old child | Middle | B40 | 25-34 | Terrace | Smoke-free home rules in place*** | 1 (father) |
| 04 | Father | 4 (age 6 months, 4, 4 and 5) | Middle | B40 | 35-44 | Semi-detached | No rules in place restricting smoking in the home | 0 |
| 05 | Father | 4 (9 years and over) | Low | B40 | 25-34 | Terrace | Smoke-free home rules in place*** | 0 |
| 06 | Father | 6 (age 2 months to 9) | High | B40 | 35-44 | Bungalow | No rules in place restricting smoking in the home | 0 |
| 07 | Father | 3 (age 5, 9 and 16) | Low | B40 | 35-44 | Terrace | Smoking is allowed in the home, but not in front of others | 0 |
| 08 | Father | 2 (age 14 and 17) | Low | B40 | 35-44 | Terrace | Smoking is allowed in the living room | 0 |
| 09 | Father | 5 age (6, 9, 15, 17 and 21) | Low | M40 | 65-74 | Bungalow | Smoke-free home rules in place*** | 0 |
| 10 | Father | 3 (age 4 months, 3 and 4) | Low | B40 | 35-44 | Other | No rules in place restricting smoking in the home | 0 |
| 11 | Adult son | Lives with parents and 14 year old siblings | Middle | M40 | 25-34 | Terrace | No rules in place restricting smoking in the home | 0 |
| 12 | Father | Partner pregnant, lives in a household which includes two nephews age 9 and 11 | Low | B40 | 25-34 | Apartment | Smoking is allowed in the home but not in front of others | 0 |
| 13 | Father | 1 (age 4 years) | Low | B40 | 25-34 | Apartment | Smoking is allowed in the living room | 0 |
| 14 | Father | 2 (age 2 months and 4) | Low | B40 | 25-34 | Apartment | No rules in place restricting smoking in the home | 0 |
| 15 | Father | 1 (age 5) | Low | B40 | 25-34 | Other | No rules in place restricting smoking in the home | 0 |
| 16 | Father | 1 (age 8) | Low | B40 | 25-34 | Apartment | No rules in place restricting smoking in the home | 0 |
| 17 | Father | 1 (age 2 months) | Low | B40 | 25-34 | Other | Smoking is allowed in the kitchen and bathroom | 0 |
| 18 | Father | 2 (age 1 month, 2) | Middle | M40 | 25-34 | Terrace | Smoke-free home rules in place*** | 0 |
| 19 | Father | 1 (age 1) | High | M40 | 25-34 | Terrace | No rules in place restricting smoking in the home | 0 |
| 20 | Father | 1 (age 13) | Middle | M40 | 25-34 | Terrace | Smoke-free home rules in place*** | 0 |
| 21 | Father | 1 (age 4) | Low | M40 | 25-34 | Apartment | No rules in place restricting smoking in the home | 0 |
| 22 | Father | 1 (age 1) | Middle | B40 | 25-34 | Terrace | No rules in place restricting smoking in the home | 0 |
| 23 | Father | 1 (age 1) | High | M40 | 25-34 | Apartment | Smoking is allowed in some rooms | 0 |
| 24 | Father | 3 (age 1, 2 and 10) | Low | B40 | 35-44 | Apartment | No rules in place restricting smoking in the home | 0 |

*Low education level = 0-6 years of formal education; middle education level = 7-12 years of formal education; high education level = More than 12 years of formal education

**B40 is the bottom 40% of Malaysia population with lower household income of < RM4850, M40 is the middle 40% Malaysia population with household income between RM4850 – RM 10,970 per month, T20 is the top 20% Malaysia population with household income of > RM10,970 per month

***Smoke-free rules were stated to be in place, but during the interview this was contradicted with references made to smoking in the bathroom (most notably) and/or the living room/bedroom
